# Pediatric Trauma Boot Camp: A Simulation Curriculum and Pilot Study

**DOI:** 10.1155/2018/7982315

**Published:** 2018-01-04

**Authors:** Ahmad Khobrani, Nirali H. Patel, Richard L. George, Neil L. McNinch, Rami A. Ahmed

**Affiliations:** ^1^Department of Pediatric Emergency Medicine, King Faisal Medical City Southern Regions, Ministry of Health, Abha, Saudi Arabia; ^2^Summa Health System, Akron, OH, USA; ^3^Department of Pediatric Emergency Medicine, Akron Children's Hospital, Akron, OH, USA; ^4^Division of Trauma, Department of Surgery, Summa Health, Akron, OH, USA; ^5^Northeast Ohio Medical University, Akron, OH, USA; ^6^Rebecca D. Considine Research Institute, Akron Children's Hospital, Akron, OH, USA; ^7^Department of Medical Education, Summa Health, Akron, OH, USA

## Abstract

Trauma is a leading cause of morbidity and mortality in infants and children worldwide. Trauma education is one of the most commonly reported deficiencies in pediatric emergency medicine (PEM) training. In this study, we describe the creation of a pediatric trauma boot camp in which trainees' basic knowledge, level of confidence, teamwork, and communication skills are assessed. The primary goal of this pilot study was to create a simulation-based pediatric trauma curriculum for PEM fellows and emergency medicine residents utilizing Kern's curricular conceptual framework. This was a pilot, prospective, single cohort, exploratory, observational study utilizing survey methodology and a convenience sample. The curriculum consisted of a two-day experience that included confidence surveys, a cognitive multiple-choice questionnaire, and formative and summative simulation scenarios. At the conclusion of this intensive simulation-based trauma boot camp participants reported increased confidence and demonstrated significant improvement in the basic knowledge and performance of the management of pediatric trauma cases in a simulated environment.

## 1. Introduction

Trauma is a leading cause of morbidity and mortality in infants and children worldwide [1, 2]. Pediatric trauma management is complex and time critical and requires the coordinated efforts of a multidisciplinary health care team [3]. Pediatric patients have several unique anatomic and physiologic differences from adult patients that require expertise for successful resuscitation [4]. Trauma education is one of the most commonly reported deficiencies in pediatric emergency medicine training [5] despite most pediatric emergency medicine (PEM) fellows receiving Pediatric Advance Life Support (PALS) and Advance Trauma Life Support (ATLS) at the beginning of their training.

One-third of medical errors leading to death in trauma patients occur in the initial emergency department evaluation and management [6]. The trauma literature reports that the ability of a team to effectively manage trauma patients during the initial evaluation depends on frequent exposure to trauma patients and quality trauma education [7]. Major pediatric trauma occurs with much less frequency than adult trauma [8]. This results in limited opportunities to develop leadership experience for pediatric emergency medicine fellows in such critical circumstances are limited.

In the ATLS course, there is little to no guidance on effective leadership, teamwork, or effective communication as a trauma team leader [9]. Additionally, there is very limited discussion about pediatric trauma [10]. The deliberate utilization of the key tenets of crisis resource management provide the foundation for the skill set necessary to be an effective member of a trauma team [11]. Medical simulation has demonstrated its effectiveness in various aspects of medical education including training interprofessional teams [12, 13], error management training [14], and acute trauma resuscitations [15]. These concepts are especially useful during a trauma resuscitation that requires the skills and expertise of an interprofessional team who may infrequently work together.

A curriculum focused on leadership, effective communication strategies, and the management of pediatric trauma patients is long overdue for pediatric emergency medicine personnel. In this study, we describe the creation of a discipline specific boot camp in which trainees' basic knowledge, level of confidence, teamwork, and communication skills are assessed. The primary goal of this study was to create a pediatric trauma curriculum for PEM fellows and EM residents.

## 2. Materials and Methods

### 2.1. Study Location and Equipment

The study was performed at on offsite simulation lab of a tertiary-care, American College of Surgeons verified Level I Trauma center, university affiliated, teaching hospital in February of 2017. High-fidelity pediatric simulators were used for all simulations. Each bay was stocked with a crash cart, Zoll AED Plus® defibrillator, Broselow® Pediatric Emergency Tape, pediatric airway equipment, pediatric vascular access equipment including an EZ-IO® intraosseus power driver and needles, and a simulated patient monitor with the ability to display the patients vital signs, diagnostic imaging, and electrocardiograms. HAL® S3005 five-year-old pediatric simulator (Gaumard®, Miami FL) was used for adolescent patients and Simulaids© Stat Manikin With Deluxe Airway Management Head (Simulaids, Inc., Saugerties, NY) was used for teenage patients. For selected cases a standardized patient pediatric actor was utilized. This was a quality assurance project that did not meet the definition of human subject research. It was exempt from institutional review board review.

### 2.2. Curriculum Development and Outline

A two-day boot camp curriculum was designed for pediatric emergency medicine fellows and emergency medicine residents on the management of traumatic injuries in pediatric patients. Kern et al.'s conceptual framework for the development of curriculum was utilized for the creation of this program [17]. The needs assessment within this framework was focused on what curricular program was most needed in the native country of the medical simulation fellow leading this study. This study served as his capstone project for his fellowship.

The curriculum consisted of a two-day, 10-hour total experience, which included a pretest confidence survey, a pretest cognitive multiple-choice questionnaire, two pretraining simulation scenarios, six additional formative simulation scenarios, a posttest confidence survey, a posttest cognitive multiple-choice questionnaire, and two final testing simulation scenarios. Simulation scenarios and curricular outline are described in Table 1. Approximately 50 hours of preparation was required from faculty leadership from the disciplines of emergency medicine, pediatric emergency medicine, trauma surgery/surgical critical care, and medical simulation for development of the curriculum, surveys, questionnaires, and simulation cases. After initial preparation, all participating staff performed a rehearsal of the trauma boot camp cases to assure that faculty and technicians had a clear understanding of the case objectives and that all necessary equipment and materials were available (5 hours). Overall, preparation and execution of the curriculum required approximately 65 hours. The goals and objectives of the curriculum and individual cases are provided in Table 2.

The curriculum had three components. The preintervention evaluation consisted of a confidence survey using a 1–5 Likert Scale on several aspects of the management of pediatric trauma. A medical simulation fellow who completed residency and fellowship training in pediatric emergency medicine developed this survey with guidance from faculty. Additionally, this fellow developed a multiple-choice test on several aspects of the management of pediatric trauma. A validated trauma team leadership evaluation tool was utilized for evaluation of critical actions during simulation testing scenarios. (Nontechnical Skills, NOTECHs) [18]. All pretraining and final testing simulated cases were recorded for evaluation and review. Two ATLS instructors (emergency medicine faculty and trauma surgery faculty) and three ATLS certified pediatric emergency medicine attending physicians evaluated the testing scenarios.

### 2.3. Participants, Faculty, and Staff

**Figure 1:**
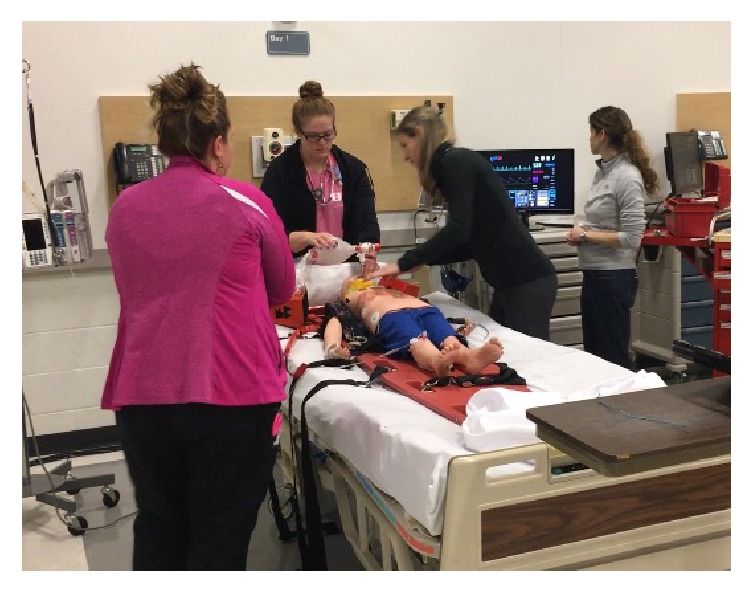
Pediatric emergency medicine fellows resuscitate a simulated patient.

Pediatric Emergency Medicine fellows from two different fellowship programs and four different emergency medicine residency programs were invited to participate. Seven emergency medicine residents and six pediatric emergency medicine fellows participated for a total of 13 participants (*n* = 13) completing the curriculum (Figure 1). Three pediatric emergency medicine attending physicians, two simulation fellows, a trauma attending, and an emergency medicine attending with fellowship training in medical simulation were present for all debriefings. Each scenario required at least one confederate nurse and one simulation technician. Some cases required the utilization of a confederate emergency medical technician and/or confederate family members.

### 2.4. Preintervention Evaluation

The preintervention stage assessed baseline confidence in several categories of pediatric trauma management.

Participants completed a 15-question confidence survey rating their confidence using a 1–5 Likert Scale [(1) Strongly Disagree to (5) Strongly Agree]. Participants also completed a 25-question cognitive multiple-choice test covering the pathologies reviewed in the simulation training cases. After completion of these two baseline assessments, the participants participated in two simultaneous patient simulations requiring two team leaders. The simulation case, a dual trauma with victim #1 (pediatric electrocution injury from a downed power line) and victim #2 (pediatric neck impalement) presenting 3–5 minutes into the case of victim #1, requires an effective team to be divided into 2 teams with 2 leaders. Two ATLS instructors (emergency medicine faculty and trauma surgery faculty) and an ATLS certified pediatric emergency medicine attending physician evaluated the testing scenarios using the validated NOTECHs scale for trauma leadership. All evaluations in this phase were summative and no feedback was provided.

### 2.5. Educational Intervention

**Figure 2:**
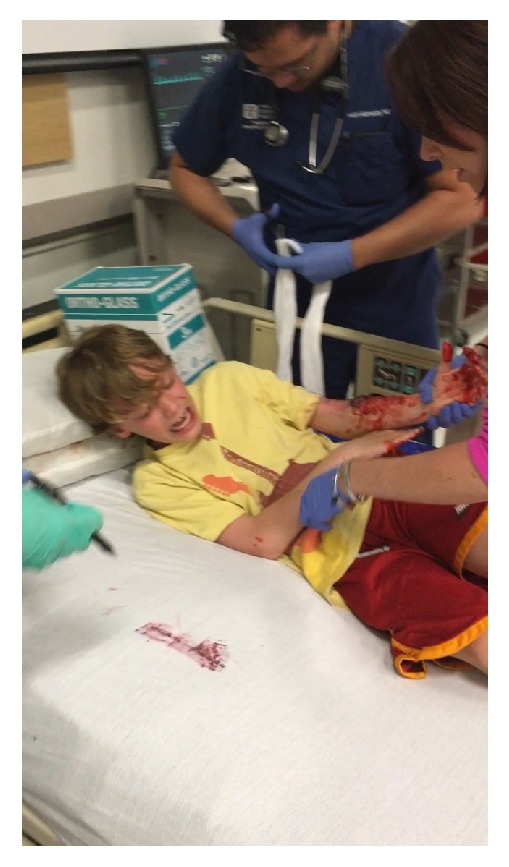
Standardized patient actor during a simulation.

The intervention phase consisted of six one-hour simulation sessions over two days. Each hour session typically consisted of a 15-minute scenario followed by a 30-minute debriefing and a succinct 15-minute PowerPoint of key points of each case. Residents were divided into two groups (6-7 participants). Two simultaneous and identical scenarios were performed in separate portions of the simulation lab (Figure 2). Four to five residents participated in a case while 2-3 residents observed. All residents were active participants in the debriefing sessions following each simulation.

### 2.6. Postintervention Evaluation

Participants underwent an identical confidence survey and multiple-choice test at the completion of the six formative simulation cases in the educational intervention stage. After the training scenarios the participants underwent another dual simulation multicasualty trauma case unique from the first case, but still providing an opportunity to test the leadership principles identified in the NOTECH's scale.

### 2.7. Postcurriculum Survey

All participants were provided a postcurriculum survey to provide feedback on areas of strength, weakness, and potential improvement of the curriculum.

### 2.8. Study Design

This was a pilot, prospective, single cohort, exploratory, observational study utilizing survey methodology and a convenience sample.

### 2.9. Data Analysis

Examination of data included summary statistics and evaluation of distribution for continuous data along with calculations of frequencies and percentages for categorical data. Testing for pre/post differences in test scores was done utilizing the paired *t*-test. Testing for pre/post differences in confidence items was done utilizing the Wilcoxon Signed Rank Test. Descriptive statistics were completed for the pre/post NOTECHS evaluations. Statistical analyses were completed using SAS 9.4/13.2© [16]. Unless otherwise noted all testing was two-tailed and evaluated at Type I Error Rate of alpha = 0.05 level of statistical significance.

## 3. Results and Key Findings

- Summary statistics for test scores are located in Table 3.
- Results and summary statistics for confidence survey questions are located in Table 4.
- Average differences for NOTECHS evaluations are located in Table 5.

The Paired *t*-Test for normally distributed continuous data provided evidence of a significant difference between (post minus pre) test scores (*p value*  ≤ 0.01), with the mean paired difference (95% CI) being 13.8% (9.2–18.5). The mean (SD) pretest score was 52.3% (10), compared to 66.2% (8.7). Refer to Table 3 for full summary statistics.

The Wilcoxon Signed Rank Test was used to compare the (post minus pre) change in responses to Likert Type items, for questions (1) through (15). Changes that were significantly different from zero included Q(1)–Q(3), Q(5)–Q(8), Q(11)–Q(13), and Q(15) (*p*-*value*  ≤ 0.03 for all). The median change for each significant item was equal to an increase of 1-2 on the Likert Scale used for each question, with corresponding interquartile ranges of (0-1) or (0–2). All changes were positive, indicating higher agreement (confidence) on the postintervention survey items. Refer to Table 4 for questions and corresponding *p values* along with paired median differences and interquartile range values, preintervention overall medians, and postintervention overall medians.

The NOTECHS scores were evaluated descriptively for each team and case (2 teams and 2 cases each) as the percentage difference in average rating from four individual raters, by domain (leadership, cooperation, communication, assessment, and situation). All percentage changes calculated represented increases from pre- to postevaluation. The smallest and largest changes in any domain also represented the greatest variability and improvement in an individual domain, which was assessment: the smallest percentage increase was 26.2% for Team 1-Case 1 and the largest percentage increase was 90.0% for Team 2-Case 1. The smallest amount of variability and improvement was observed in leadership, with a min/max change of 27.5% for Team 1-Case 1 and 48.9% for Team 2-Case 2. See Table 5 for complete list of changes by team, case, and domain.

## 4. Discussion

This two-day curriculum resulted in increased self-confidence, knowledge of pediatric trauma management, and performance in a simulated environment. The improvement in basic knowledge likely reflects the benefits of well-conducted expert debriefings with reinforcement of key teaching points followed by focused didactic PowerPoint presentations on the selected pathology of each simulated case. After completing the curriculum, learners demonstrated statistically significant improvement in reported self-confidence in several areas, most importantly noting increased comfort in the performance of primary and secondary exams, role delegation, effective closed loop communication, disposition of pediatric trauma patients, managing pediatric trauma airways, and the management high voltage electrical injuries (*p value* < 0.01 for all). The scenarios were setup keeping deliberate practice methods in mind [19]. This allowed repeated reinforcement of critical teaching points in each case with immediate corrective feedback by content experts providing expert level debriefing under the guidance of the simulation faculty.

Interestingly, our study did not show significant increases in self-confidence in several areas. There was no significant improvement in confidence in the determination of the Glasgow Coma Scale (GCS) to guide to the care of the patient. Although we consider the GCS a basic concept for emergency medicine trainees, we only provided limited time during cases and debriefings to discuss this concept. Additionally, there was no increase in confidence in orthopedic splinting/reduction and, that too, may be attributable to limited postsimulation discussion during debriefing. Many of the learners report that at their respective centers the orthopedic staff typically takes over these procedures in the emergency department providing limited clinical exposure, suggesting more hands-on training in this skill. Another area demonstrating no statistically significant improvement in confidence was ordering appropriate diagnostic testing for pediatric trauma patients. This particular area started with a high preconfidence level and stayed high. Most surprisingly, there was no significant improvement in pediatric FAST exam confidence despite every case necessitating the performance of a FAST exam. This suggests more extensive training is needed in this critical skill set in pediatric trauma management. In future iterations of this boot camp, this will be a standalone hands-on skill station added to the curriculum taught by faculty to reinforce previous training.

To assess pediatric trauma management, teamwork skills participants were divided into two distinct teams throughout the curriculum with each team containing PEM fellows and EM residents of different training levels and programs. Our results demonstrate significant increases in all 5 behavioral domains of teamwork performance for both teams (see Table 5). The highest behavioral domain increase demonstrated during simulations was in assessment and decision-making. This was manifested by an improvement in the ability of both teams to appropriately complete the primary and secondary surveys, consistently share a mental model with the team, and prioritize critical management steps. There was also clear improvement in closed loop communication by the trauma team leaders. In addition, despite multiple preplanned challenges embedded in each simulation case (disruptive families, multiple casualties, limited staff availability, etc.), both teams demonstrated improvement in proceeding in a systematic fashion while minimizing distractions. By the end of the curriculum, all team members were able to assign roles, utilize clear closed loop communication, and demonstrate effective management as team leader. Despite our best efforts, every learner in the study did not get an opportunity to serve as a team leader during the execution of the ten cases. This may have affected the confidence scores for those not given the opportunity to function as a team leader.

Functioning as an effective trauma team and utilizing CRM principles in acute pediatric trauma settings require continued simulation training. High-fidelity medical simulation allows for education and evaluation of trauma team performances through direct feedback and debriefing in a safe learning environment with expert faculty. Furthermore, these types of encounters help improve team dynamics [20]. There are previously published studies with similar curricula. Ortiz Figueroa et al. developed a one-day boot camp for emergency medicine interns focused on trauma management [9]. Their training included hands-on skills, didactics, and simulation scenarios and it similarly demonstrated an increase in confidence in CRM principles of role delegation, leadership, and performance of the primary and secondary survey. However, this study demonstrated no significant improvement in overall teamwork and leadership performance. Additionally, a similar three-day curriculum published by Miyasaka et al. focused on trauma, surgical, and critical-care scenarios that included hands-on skills, didactics, and simulation scenarios [21] that resulted in increased confidence for surgical residents.

Pediatric trauma continues to be the leading cause of morbidity and mortality in infants and children worldwide [22]. Our study and previous similar studies demonstrate the importance of curriculum as a fundamental tool in pediatric trauma management education, especially as this is a commonly reported deficiency in pediatric emergency training programs [5]. Despite all the learners having completed the ATLS course at the beginning of their training, this program demonstrated significant educational benefit to this cohort of learners. This is especially valuable in countries like Saudi Arabia, where there is little trauma training outside of trauma centers.

In the Kingdom of Saudi Arabia (KSA), patients sustaining severe injuries in motor vehicle collisions are more likely to die in comparison to similarly injured patients in the US [23]. Trauma is the leading cause of death in the KSA [24]. Currently, the healthcare system in KSA is lacking an appropriate number of tertiary-care trauma centers to serve the needs of the entire country [24]. Only two facilities are designated as pediatric trauma centers with resuscitations led by pediatric emergency medicine attending physicians [24]. All pediatric trauma patients get transported to the closest trauma center (bypasssing all pediatric facilities) and the vast majority are initially managed by adult emergency medicine physicians, limiting the exposure of PEM fellows in the management of such patients. Curricula, such as the one described here, are critical to the preparation and continued readiness of pediatric emergency medicine faculty and staff.

Our pediatric trauma boot camp curriculum was well received by participants as supported by feedback from a postcurriculum survey. Positive feedback included life-like simulation scenarios including a high-pressure environment, multidisciplinary debriefings, and succinct reviews of critical management points. Learners also reported that faculty feedback and focused PowerPoint presentations were highly beneficial in improving their knowledge, self-confidence, and leadership and communication skills. Learners commented the need for increased practice opportunities through simulation for low frequency procedures (FAST exam, pericardiocentesis, etc.). Additionally, residents did not feel overwhelmed by the amount of material covered.

## 5. Limitations

Our study has several limitations. First, our study was a small pilot study with a small sample size of learners from three community-teaching hospitals. We believe a randomized design study with a control group of traditionally trained emergency medicine residents and pediatric emergency medicine fellows compared to an experimental group of simulation trained learners would have provided more powerful data. Second, this study was done one time over two days and repeating this boot camp over several sessions would both increase the number of learners in the study and check its reproducibility. Finally, the faculty also served as the graders of the summative simulations via video review of all cases and were not blinded to the pre- versus post-scenario training performances, potentially introducing some bias.

## 6. Conclusion

At the conclusion of this intensive simulation-based trauma boot camp participants demonstrated significant improvement of the basic knowledge, confidence, and performance in the management of pediatric trauma cases. Our boot camp curriculum offers educators a unique framework that they can apply to their own training program as a foundation for effective leadership and teamwork training for the management of pediatric trauma.

## Figures and Tables

**Table 1:** Curricular outline.

Day one	Day two
Day one introduction	Day two introduction
Confidence survey and multiple choice questionnaire	Scenario 6

Test Cases Scenario 1 Scenario 2	Scenario 7

Scenario 3	Scenario 8

Scenario 4	Test cases Scenario 9 Scenario 10

Scenario 5	Confidence survey, multiple choice questionnaire, and boot camp evaluation

**(a):** 

Goals
(1) Demonstrate the qualities and behaviors of an effective pediatric trauma team leader (2) Demonstrate the qualities and behaviors of an effective pediatric trauma team member (non-leadership role) (3) Demonstrate utilization of crisis resource management principles (4) Demonstrate effective primary and secondary surveys with initiation of immediate life and limb saving interventions

**(b):** 

Description of simulation cases with objectives
*#1 and #2: dual trauma (test cases)*
(a) Electrical Injury
(i) Demonstrate effective management of high-voltage electrical injuries (including dysrhythmias, rhabdomyolysis, and compartment syndrome)
(ii) Demonstrate effective pain management and fluid resuscitation in burn victims
(iii) Demonstrate an understanding of the criteria to transfer to a burn a center
(iv) Demonstrate effective closed loop communication
(b) Neck impalement
(i) Prepare for and obtain a difficult airway
(ii) Demonstrate effective management of an impaled foreign object in the neck
(iii) Demonstrate effective needle decompression followed by emergent placement of a chest tube for tension pneumothorax
(iv) Demonstrate effective closed loop communication
*#3: burn with trauma (jump out of a burning building)*
(i) Identify and manage airway compromise in a burn victim
(ii) Recognize and manage full thickness burns of the chest
(iii) Demonstration of escharotomy for circumferential chest/back burns
(iv) Demonstrate proper fluid resuscitation in a major burn patient (e.g. Parkland)
(v) Identify carbon monoxide and cyanide exposure as potential diagnoses
*#4: abdominal and Pelvic Trauma (motor vehicle collision)*
(i) Demonstrate effective management of a severe head injury
(ii) Demonstrate ability to perform a FAST exam
(iii) Demonstrate ability to apply a pelvic binder
(iv) Demonstrate ability to utilize the massive transfusion protocol
*#5: building collapse with compartment syndrome*
(i) Recognize and treat crush syndrome
(ii) Demonstrate effective use of tourniquets
(iii) Demonstrate understanding of team safety above patient care
*#6: gunshot with penetrating neck, chest, and abdominal injuries *
(i) Demonstrate ability to prepare and acquire a difficult airway
(ii) Recognize potential for intra-abdominal pathology with penetrating injuries to the chest
(iii) Demonstrate ability to emergently place a chest tube
(iv) Demonstrate ability to utilize the massive transfusion protocol
*#7: blunt chest trauma with pericardial effusion*
(i) Demonstrate ability to manage blunt chest trauma
(ii) Demonstrate ability to execute FAST exam
(iii) Demonstrate ability to perform emergent pericardiocentesis
*#8. Neurogenic shock*
(i) Identify neurologic deficits in the primary survey
(ii) Recognize signs of neurogenic shock secondary to spinal injury
(iii) Demonstrate ability to rule out other cause of hypotension before initiating treatment for neurogenic shock
(iv) Demonstrate ability to manage neurogenic shock including utilization of vasopressors and cardiac pacing
*#9 and #10: dual trauma (test cases)*
(a) Motor vehicle collision with intracranial hemorrhage
(i) Demonstrate ability to obtain a difficult airway
(ii) Demonstrate ability to manage a severe head trauma with signs of herniation
(iii) Demonstrate effective closed loop communication
(b) Lower limb amputation with hemorrhagic shock
(i) Demonstrate effective exsanguinating hemorrhage control techniques including the utilization of tourniquets
(ii) Demonstrate appropriate management of a distal extremity amputation
(iii) Provide appropriate analgesia for an unstable conscious victim
(iv) Demonstrate effective closed loop communication

**Table 3:** Test scores data: summary statistics and paired *t*-test results.

	*N*	Mean	Std Dev	Min	Max
Pre score	13	52.3	10	36	72
Post score	13	66.2	8.7	52	84
Paired difference^*∗*^	13	13.8	7.8	4	32

^*∗*^Paired *t*-test: *p-value* < 0.001. 95% CI of mean diff: (9.2–18.5). See [16].

**Table 4:** Confidence survey data: pre-post difference.

Confidence questions	*p value*	Median (paired difference)	Lower quartile	Upper quartile	Overall median (pre)	Overall median (post)
(1) I know how to effectively perform primary and secondary surveys during a pediatric trauma resuscitation.	*0.008*	*1*	*0*	*1*	*4*	*5*
(2) I feel confident I know when a pediatric trauma patient should be intubated in the trauma bay.	*0.008*	*1*	*0*	*1*	*3*	*4*
(3) I am confident I could intubate a pediatric trauma patient using in line immobilization.	*0.014*	*1*	*0*	*2*	*3*	*4*
(4) I know the Glasgow Coma Scale (GCS) and use it to guide the care of a patient.	0.375	0	0	1	4	4
(5) I understand when a pediatric trauma patient is considered hemodynamically unstable.	*0.031*	*1*	*0*	*1*	*4*	*5*
(6) I am confident I could be the primary team leader during pediatric trauma activation and effectively lead my team.	*0.004*	*1*	*0*	*2*	*3*	*4*
(7) I am confident I can effectively delegate roles for the members of my team.	*0.008*	*1*	*0*	*2*	*3*	*5*
(8) I am confident I can consistently provide orders in a closed loop fashion during a pediatric trauma resuscitation.	*0.009*	*1*	*1*	*1*	*3*	*4*
(9) I am confident I can order appropriate diagnostic tests for a pediatric trauma patient.	0.109	1	0	1	4	4
(10) I am confident I can perform a pediatric FAST exam.	0.063	0	0	2	3	4
(11) I am confident I know when to transfer a patient to the operating room vs. keeping them in the emergency department for frequent reevaluation.	*0.014*	*1*	*0*	*2*	*3*	*4*
(12) I am confident I know when to transfer a pediatric trauma patient to a trauma center.	*0.008*	*1*	*0*	*1*	*4*	*4*
(13) I am confident I can manage a burn patient adequately and know the indications for transfer to a burn center	*0.001*	*1*	*1*	*2*	*3*	*4*
(14) I am confident I can adequately reduce/splint fractures in a trauma setting.	0.219	0	0	1	3	3
(15) I am confident I can manage a high voltage electrical injury patient.	*<0.001*	*2*	*1*	*2*	*2*	*4*

See [16].

**Table 5:** NOTECHS data: pre-post, percentage improvement in average ratings by domain.

	Leadership	Cooperation	Communication	Assessment	Situation
Team 1-Case 1	27.5	27.7	41.0	26.2	31.1
Team 1-Case 2	65.1	59.0	72.9	62.5	51.0
Team 2-Case 1	34.6	51.0	59.2	90.0	69.4
Team 2-Case 2	48.9	62.1	72.9	74.4	65.1

See [16].
